# Pre-clinical delivery of gene therapy products to the cerebrospinal fluid: challenges and considerations for clinical translation

**DOI:** 10.3389/fnmol.2023.1248271

**Published:** 2023-08-17

**Authors:** Ernesto A. Salegio, Kelli Hancock, Stephanie Korszen

**Affiliations:** ClearPoint Neuro Inc., Solana Beach, CA, United States

**Keywords:** cerebrospinal fluid, intrathecal, cisterna magna, gene therapy, central nervous system, primates, adeno-associated virus

## Abstract

While the majority of gene therapy studies in neurological indications have focused on direct gene transfer to the central nervous system (CNS), there is growing interest in the delivery of therapeutics using the cerebrospinal fluid (CSF) as a conduit. Historically, direct CNS routes-of-administration (RoAs) have relied on tissue dynamics, displacement of interstitial fluid, and regional specificity to achieve focal delivery into regions of interest, such as the brain. While intraparenchymal delivery minimizes peripheral organ exposure, one perceived drawback is the relative invasiveness of this approach to drug delivery. In this mini review, we examine the CSF as an alternative RoA to target CNS tissue and discuss considerations associated with the safety of performing such procedures, biodistribution of therapeutics following single administration, and translation of findings given differences between small and large animals. These factors will help delineate key considerations for translating data obtained from animal studies into clinical settings that may be useful in the treatment of neurological conditions.

## 1. Introduction

Strategies to deliver therapeutics to the central nervous system (CNS) can be classified into three main categories: (a) those administered systemically with the goal of penetrating through the blood–brain barrier (BBB), (b) those administered focally via direct intraparenchymal delivery, and (c) those administered into the cerebrospinal fluid (CSF) compartment. Each approach poses different challenges for the field of drug delivery, and the focus herein is to highlight current literature regarding the use of CSF as an alternative route-of-administration (RoA) to target CNS tissues. Obvious benefits of delivering into the CSF over systemic administration include reduced drug exposure to peripheral organs, significantly smaller drug volume required, and reduced invasiveness compared to intraparenchymal delivery. These benefits are particularly relevant to gene therapy products, specifically adeno-associated viral (AAV) vectors, as AAVs are expensive to manufacture, require high doses when administered systemically, and are known to continue expressing the gene(s) of interest many years after a single administration (George et al., 2020).

Within this review, we discuss challenges associated with CSF delivery of AAV products when targeting CNS tissue, including BBB penetration, limitations with reproducibility, expected off-target effects, maximization of global drug spread within the CNS, translation across animal species, and clinical considerations.

## 2. Challenges to overcome in the CNS

### 2.1. Blood–brain barrier (BBB) penetration

The BBB is a selective endothelial structure that functions to maintain separation between the bloodstream and CNS but poses a unique challenge to achieving drug delivery to the brain and spinal cord. Non-invasive approaches, such as administration of drugs systemically, have shown limited therapeutic benefit due to the poor penetrability of drugs across the BBB and into the CNS (Zlokovic, 2008). This type of selective trafficking is size-dependent (<400 Da) and excludes more than 98% of small molecules and 100% of large molecules (Pardridge, 2007). This is of particular concern in younger populations with CNS disorders in which the BBB is intact, direct intraparenchymal procedures are more challenging, and systemic dosing poses a greater risk. The integrity of the BBB may be impacted in older adults, given a correlation between BBB leakage in anatomical regions sensitive to age-related deterioration – a process described as a “normal physiologically aging phenomenon” (Verheggen et al., 2020).

Recent technological advances in AAV production have made it possible to create a new generation of AAV vectors (i.e., hybrids) that are useful for many clinical applications. One such hybrid that has gained considerable interest, a double-stranded AAV serotype 9, has demonstrated an ability to surpass the transduction efficiency of its single-stranded counterpart (Duque et al., 2009) and better bypass the BBB when administered systemically (Foust et al., 2009). Further, Gray et al. (2013) showed that the AAV2.5 serotype, a hybrid of AAV2 and AAV1, was also able to cross the ependymal barrier when delivered into the ventricles and exhibited similar transduction patterns as AAV9 in the brain and spinal cord – with significantly less transduction in the spleen than AAV9. Intraparenchymal (IP) delivery into the CNS can avoid these complications by directly administrating drugs into CNS tissue, thus avoiding the BBB. However, IP delivery requires a neurosurgical procedure and is a more invasive approach than intravenous or CSF delivery. Nonetheless, IP allows direct access to neuronal relays, which is advantageous in certain applications. For instance, gene products delivered into highly interconnected regions in the non-human primate (NHP) brain can facilitate robust AAV distribution following a single injection (Kells et al., 2009). Concerns regarding the safety and tolerability of such procedures for CNS disorders like Niemann-Pick and Parkinson’s disease have already been addressed, but these remain challenging procedures (Salegio et al., 2010; San Sebastian et al., 2012). Conversely, administering drugs into the CSF offers a less invasive alternative that allows for global gene transfer to CNS tissue, brain, and spinal cord using AAV7 and AAV9 (Samaranch et al., 2011, 2013), and it is a clinically relevant approach proven to achieve widespread distribution of therapeutics within the CNS.

### 2.2. Limitations with reproducibility

Selection of the appropriate AAV serotype and animal species is important, and the ultimate pattern of biodistribution will be determined by the RoA utilized. Different serotypes vary in their tropism, their ability to infect cells, and outcomes can be species-dependent (San Sebastian et al., 2013). For instance, Gray et al. reported that AAV serotypes 2, 4, and 5 did not effectively transduce neurons in the brain parenchyma when administered intrathecally in NHPs (Gray et al., 2013). Similarly, improved distribution has been reported after cisterna magna (CM) delivery using AAVrh.10 when compared to intracerebroventricular (ICV) or intraparenchymal (Rosenberg et al., 2018). Sorrentino et al. (2016), studied the tropism of multiple AAV serotypes (1, 2, 5, 7, 9, rh.10, rh.39, and rh.43) after CM injection in pigs, and observed significantly different patterns of cell transduction throughout various parts of the CNS. In their study, AAV9 transduced both glia and neurons, was the only serotype to transduce spinal motor neurons, and resulted in the best transgene expression along the entire CNS. Further, other reports in rodents indicate that delivery of AAV8 into the CSF can transduce the spinal cord and dorsal root ganglia (DRG) (Storek et al., 2008). Snyder et al. (2011), concluded that lumbar intraparenchymal injection of AAV9 in mice was superior to that of AAV1, AAV6, and AAV8 and produced localized cell body transduction in the lumbar region, whereas AAV6 performed best via intrathecal delivery, resulting in widespread gene expression.

Transport and binding of AAVs are also important considerations. AAV2 is known to be transported anterogradely, and it seems that this phenomenon is not species-specific (Ciesielska et al., 2011). Conversely, other AAVs, such as AAV6, almost exclusively undergo retrograde transport (Salegio et al., 2013). The exact mechanism regulating this type of AAV transport remains unclear, although it is known that directionality in axonal transport may be partially mediated by variations in capsid sequence, and possibly by receptor-cell interaction (Nonnenmacher and Weber, 2012). The tropism of each AAV serotype is associated with its capsid amino acid sequence and/or receptor binding affinity, which could account for the strong neuronal tropism of AAV2 and the glial/neuronal tropism of AAV9. In fact, AAV9 shares 82% capsid homology with AAV2, which explains its tropism for neurons (Daya and Berns, 2008). Modifying AAV2 by altering its binding affinity has also yielded interesting results, with extensive transduction spread reported following a single infusion in the NHP brain (Naidoo et al., 2018).

### 2.3. Expected off-target effects

When administering drugs into the CSF biodistribution will be mediated by the kinetic flow of CSF (Khani et al., 2022) and therefore contact with “off-target” surrounding structures is likely to occur. One unintended target following CSF administration is the DRG. Studies suggest that transgene overexpression can lead to cellular toxicity in the cells that express the most transgene protein (Hordeaux et al., 2020a) and age at the time of injection (as well as vector dose) had a significant impact on pathology severity, but RoA and gender did not (Hordeaux et al., 2020b). Vector-induced toxicity has been observed in the DRG of NHPs following AAV administration into the CSF (Hinderer et al., 2018; Hordeaux et al., 2018; Perez et al., 2020) and this creates concern for the potential implications of a similar toxicity occurring in humans. Although these NHPs presented asymptomatically, it remains unclear what side effects result from this type of transduction. Authors also noted that DRG pathology was greater in NHPs dosed through the CSF than in those dosed intravenously. RoA and DRG transduction has been corroborated in multiple studies utilizing various AAV serotypes like 2.5, 7, and 9 (Gray et al., 2013; Samaranch et al., 2013; Hordeaux et al., 2019), and the RoA played an important role in the level of transduction, with intracisternal administration showing lower efficiency than injections into the lumbar space (Gray et al., 2013). RoA and overall physical positioning of the patient to alter CSF flow after injection has also been noted to play a strong role in the final distribution of the transgene within the CNS and is further discussed below. Another important consideration when administering AAVs into the CSF is the off-target transduction of peripheral organs, as explored by several studies following CM and/or intrathecal administration (Samaranch et al., 2013; Hinderer et al., 2014, 2018). Reassuringly, the level of protein expression in the liver and spleen have been reported to contain low transgene expression (Gray et al., 2013).

## 3. Clinical implications

### 3.1. Maximizing global spread of therapeutics

**FIGURE 1:**
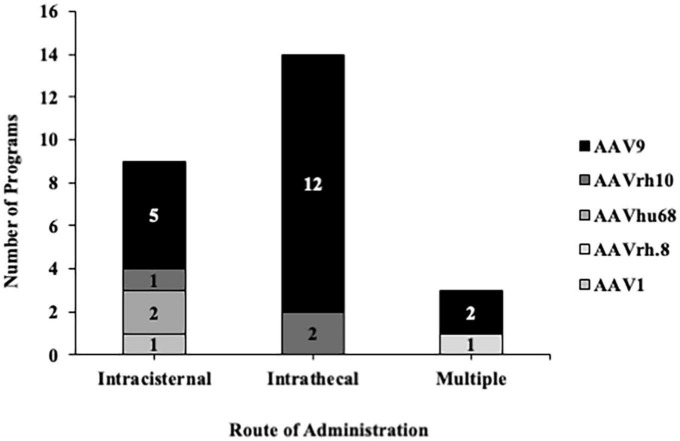
Adeno-associated vector serotypes used in clinical trials for intrathecal and intracisternal administration. A search on clinicaltrials.gov using the terms “cistern, intrathecal + AAV, ICM” indicated that the most commonly used anatomical entry point for delivering therapeutics into the CSF is the intrathecal space. Regardless of the entry point, most of these trials implemented AAV9 as the backbone.

As aforementioned, selecting the appropriate RoA and optimizing patient positioning are crucial to circumvent the need for AAV re-administration and to maximize the global spread of a therapeutic. Previous data indicates that injection into the cisternal lumen is the best preclinical RoA for broad biodistribution to the brain and spinal cord (Samaranch et al., 2011, 2013; Hinderer et al., 2014; Katz et al., 2018; Ohno et al., 2019). However, this route is less commonly performed clinically due to needle proximity to the brainstem and cerebellum. Nonetheless, in a recent review, authors describe ongoing work to improve methods for performing injections into the cisternal lumen and lumbar space, dating back to 1920 (Lutters and Koehler, 2020). While improvements have been made to the clinical workflow over the course of the past century, one key element that has been relatively ignored is the lack of commercially available devices optimized for CSF delivery – particularly those that could be used to target regions proximal to motor-sensitive anatomy like the CM. Regardless, a century later there is still a strong demand for clinical translation, and a search of clinical trials involving AAVs delivered into the CSF indicates that at least 26 trials leverage intracisternal and/or intrathecal RoAs (Figure 1). Of the 26 trials, the most commonly used serotype was AAV9, and one trial resulted in a commercially approved product (Zolgensma^®^).

The physical positioning of the subject is an important consideration. Studies placing subjects in the Trendelenburg position, whereby the subject is tilted 15–30° with their feet elevated above their head during and after the infusion, reported favorable biodistribution (Meyer et al., 2015). However, there is variability between studies and implementation of the Trendelenburg technique still requires thorough investigation (Hinderer et al., 2018). This is an area of continued exploration, with more consistent animal-to-animal results recently reported (Castle et al., 2018). Another approach to maximize biodistribution involves combining multiple CSF RoAs, as demonstrated by Ohno et al. (2019), whereby direct comparisons were made following single ICM, lumbar intrathecal, and ICV, versus combined ICM and lumbar injections. Of these routes, the greatest biodistribution throughout the CNS was observed after combined ICM and lumbar injections.

Another element to overcome, particularly in the field of gene therapy, is avoidance of pre-existing neutralizing antibodies (Nabs) to AAV. Two important factors that need to be considered when administering a viral vector, especially if re-administration is sought, are: the presence of Nabs, and immune cell responses that may affect gene expression and global distribution. In humans, seropositivity to AAV is prevalent (Blacklow et al., 1967; Chirmule et al., 1999; Boutin et al., 2010), although it is not closely correlated with white blood cell proliferation (Chirmule et al., 1999). Authors suggest that the AAV humoral response may be T-cell independent due to lymphoproliferative responses to the AAV capsid, and not to a particular transgene. Conversely, there is evidence indicating that intra-striatal injections in rodents yielded an immune response against the AAV capsid, but re-administration of the transgene using a different AAV capsid did not lead to loss of gene expression or immune activation (Peden et al., 2009). Priming the CNS using two different capsids may be of interest in future indications, particularly those implementing MRI-guided delivery, because transgene expression may still be regulated by the immune system – even in the absence of AAV leakage into the CSF during intraparenchymal administration. One such study showed evidence of this but used a green-fluorescent protein (GFP) tag (Salegio et al., 2022). More research on this is therefore warranted, as it is well understood that introduction of foreign transgenes like GFP can trigger a cell-mediated immune response (Samaranch et al., 2013).

### 3.2. Translation across animal species

A key translational challenge when using small animal species is the up-scaling effect to humans, particularly in relation to differences in drug volumes, anatomy, tissue properties, and biodistribution. Consider that the adult human brain weighs approximately 1,500 g and contains 86 billion neurons, while the adult macaque brain weighs 87 g and contains 6 billion neurons, and the mouse brain weighs 0.4 g and contains 70 million neurons (Van Essen et al., 2019). Therefore, when calculated in terms of physical size, the human brain is approximately 3,750 times larger than a mouse brain, and about 17 times larger than that of an NHP. Brain volume is one important consideration for direct intraparenchymal delivery and simple multiplication cannot dictate how much drug volume should be administered focally in different animal species. Rather, deciding upon infusion volumes requires a more in-depth understanding of NHP and human neuroanatomy, immunology, and functionality – further informing the need for effective translation to clinical applications (Courtine et al., 2007).

Similarly, differences in CSF volumes and turnover rates must also be considered when translating results between species, particularly those involving CSF as a conduit (Table 1). For instance, human CSF turnover rates are relatively similar to that of NHPs at 4–5 volumes per day, but in rodents CSF turnover occurs at least 11–14 times per day, and their total CSF volume is significantly smaller than that of humans or NHPs. The scaleup from the small animal CSF volume to that of humans is even greater than that of brain volume, with a 4,000-fold difference. In addition to considering CSF volume and turnover rates when evaluating large animal models, it is also important to compare diffusion rates and composition of CSF between species. In one study, a differential cell count found a comparable ratio of lymphocyte to monocytoid cells in both cynomolgus monkeys and beagle dogs (Ballesteros et al., 2020), but a significantly lower ratio in Gottingen minipigs. This same study also assessed diffusion rates after an intrathecal injection of a contrast agent and showed that beagle dogs had the longest diffusion times with the infusate covering the most distance, followed by NHPs, and lastly minipigs. Similarly, intrathecal administration can result in gene expression limited to the spine in rodents (Storek et al., 2006; Towne et al., 2009; Vulchanova et al., 2010; Snyder et al., 2011; Gray et al., 2013), but a more robust spread in larger animals (Federici et al., 2012). These interspecies discrepancies should be considered when developing clinical protocols and support the utility of large animal models. Researchers must not only consider how to “scaleup” the volume to administer, but also target selection, RoA, delivery approach, dosage, biodistribution, safety, toxicity, and tropism. As eloquently stated by Crystal, “humans are not simply large mice” (Crystal, 1995). Therefore, selection of the appropriate preclinical species will continue to remain an area of focus in the field of translational gene therapy.

**TABLE 1:** Comparative summary of inter-species differences in total CSF volume, production and turnover rate between humans, cynomolgus macaques, and rodents.

Species	Total CSF volume (mL)	Production rate (μl/min)	Turnover rate (Volume/Day)
Human	150–160^1^	300–600^1^	4^1^
NHP (Cyno)	11.6^2^	29–41^3^	4–5^4^
Rat	0.196^1^	1.48^1^	11^1^
Mouse	0.04^1^	0.325^3^	12–14^4^

Findings summarized from four previous studies highlight important considerations when scaling up from human to rodents and how these vary between animal species (Johanson et al., 2008; Casaca-Carreira et al., 2018; Fowler et al., 2020; Sullivan et al., 2020). ^1^ Johanson et al. (2008). ^2^ Sullivan et al. (2020). ^3^ Casaca-Carreira et al. (2018). ^4^ Fowler et al. (2020).

## 4. Discussion – future directions

The paramount aims of any gene therapy is to create vectors using only the necessary machinery to achieve efficient transduction levels, minimize any host-immune response to the vector or transgene, and increase biodistribution as needed. High gene expression can result in inflammation and/or clearance of infected cells and humans may be naturally infected with AAV serotypes 2, 3, and 9 (Blacklow et al., 1967; Boutin et al., 2010), so screening of populations prior to receiving any type of treatment is important in any clinical protocol. Further, regardless of whether a gene therapy is destined for children or adults, exclusion criteria such as prior infections should be a determining factor. For instance, previous clinical observations have reported development of a leukemia-like disorder accompanied by a strong T-cell response, which may have been related to a chickenpox infection (Marshall, 2002). Currently, most clinical trials involving CSF administration are still leveraging AAV9-based investigational products, but the increasing number of publications involving CSF delivery of novel hybrids with promising preclinical data suggests that there will be a more diverse range of AAVs in future intrathecal and intracisternal trials. While there will still be instances when localized drug-CNS interaction to either the brain or spinal cord alone is preferred (Lutters and Koehler, 2020), CSF delivery enables more global widespread distribution and may be performed as a routine procedure.

Importantly, clinical safety guidelines regarding needle penetration into the CSF compartment have denoted low complication rates, with the most commonly reported side effects including headache or back pain (Engelborghs et al., 2017). Regardless of the safety profile, repeatability of the dosing is a key concern. Even under image-guidance, the success rate of delivering a full therapeutic payload into the CSF compartment in NHPs was demonstrated to be only 67% (Ohno et al., 2019). One potential source of variability when delivering drugs into the CSF may be the delivery device, with the most common being a “standard” stainless steel spinal needle, as these are readily available, and few alternatives exist. For any commercially available spinal needle, delivery of AAV products would be considered off-label usage, as it does not fall within the currently approved intended use for this category of devices. Variations in dosing when using a spinal needle may be caused by slight movement of the needle tip during connection of the syringe, minor accidental repositioning of the animal, and/or human error due to the size constraints of the CSF space. This may be mitigated by a novel device with an anchoring system to ensure that the device remains within the CSF space for the duration of the procedure. In addition to reliability for bolus dosings, such a device would enable longer term infusions, which are currently not feasible with a handheld spinal needle. With the increase in programs leveraging CSF delivery and the importance of accurate and repeatable dosing, industry must continue to improve upon available devices to meet the growing need for preclinical studies, clinical trials, and eventually commercial approval.

## Author contributions

ES wrote the main portions of the manuscript. KH and SK made significant contribution writing sub-sections. All authors reviewed and edited contents of the manuscript and approved it for publication.
